# Western Dental Society

**Published:** 1865-09

**Authors:** 


### WESTERN DENTAL SOCIETY.

The Annual Meeting of the Western Dental Society was held at Saint Louis, commencing January 18th, 1865, and continuing two days.

The Society assembled at 12 M., in room of Board of Public Schools. In the absence of the President, the Society was called to order by Second Vice-President, W. H. Eames.

The proceedings of the last Annual Meeting were read and approved.

Members from abroad not having yet arrived, Society adjourned till 2½ P. M.

The Society met at 2½ P. M., Prest. T. P. Abell, of Chicago in the Chair.

The Committee on Microscopic Preparations, appointed at the last meeting, asked for and obtained further time. On motion, Dr. Allport was added to the committee.

The Committee to whom the subject of the free tuition of a dental student was referred, reported no applicant for the gratuitous scholarship. On motion the committee was discharged.

The regular order being the election of officers, ballotings were had, resulting in the choice of the following officers for the ensuing year:

President—T. P. Abell, of Chicago; First Vice-President—C. W. Rivers, of Pittsfield; Second Vice-President—W. H. Eames, of St. Louis; Recording Secretary and Treasurer—C. W. Spalding, of St. Louis; Corresponding Secretary—J. W. Ellis, of Chicago.

Executive Committee—W. W. Allport, J. W. Ellis, J. C. Fuller, of Chicago, H. E. Peebles, and W. N. Morrison, of St. Louis.

The Society also selected the following Delegates to the American Dental Association: C. W. Rivers, S. L. Edwards, M. McCoy, J. Comstock, J. W. Ellis, G. S. Miles, D. W. Perkins, S. H. Anderson, Charles Knower and H. N. Lewis.

On motion of Dr. I. Forbes, it was

*Resolved*, That the Corresponding Secretary be empowered to set forth in his communications to the members the necessity of attending the Annual Meetings of the Society, and if absent, to request them to report by letter.

On motion of Dr. Peebles, it was

*Resolved*, That the Corresponding Secretary be requested to issue a circular letter to the members of this society, and other dentists, residing in the States of Iowa, Illinois, Michigan, Wisconsin and Missouri, notifying them of the time and place of the next Annual Meeting; and intimating that the Western Dental Society may at that meeting be changed to that of the Missouri and Illinois State Society, and to cordially invite them to be present at the meeting.

On motion of Dr. Spalding, a Committee of Three was created to report at the earliest practicable time on the most feasible plan for obtaining funds for the purchase of a strictly first-class microscope.

An interesting discussion ensued, which was participated in by most of the members present. It was believed that the procuring of such an instrument might result in the solution of certain unsolved questions in dental histology.

The Chair appointed Drs. Spalding, Allport and Forbes that Committee.

On motion the President was added to committee.

Adjourned to 7 P. M.

The Society met pursuant to adjournment. President in the chair.

On motion of Dr. Peebles, the annual dues were restored, for the purpose of increasing the fund in the treasury, with a view to the purchase of a microscope by the society.

On motion of Dr. McCoy, the dues were fixed at $3 per annum.

On motion of Dr. Spalding, clinics was made the special order for eleven o'clock to-morrow.

Adjourned to ten o'clock to-morrow.

---

THURSDAY, January 19, 1865.

The Society met pursuant to adjournment. President in the Chair.

The use of the mallet in filling teeth was discussed by Drs. Eames, Spalding, Peebles, Blake and others. Its employment was unanimously approved by those who had given it a fair trial, one of the speakers declared that if this meeting of the Society should result in nothing more than to induce all the members present to adopt its use, they would be amply compensated for the time spent in attending the meetings.

The discussion continued until the hour of eleven o'clock, when the special order was taken up, viz: clinics; illustrating the use of the mallet, which occupied the rest of the morning session.

The Society met at 2½ P. M. President Abell in the Chair.

The next Annual meeting was fixed to take place at Springfield, Illinois, on the first Wednesday in January, 1866.

Dr. Ellis spoke of the difficulty he experienced in keeping cavities in the teeth dry while introducing the filling. Had sometimes been compelled to perform what might be called submarine operations.

Dr. McCoy met with the same difficulty, which he had not always succeeded in overcoming, but had sometimes been compelled to perform moist operations.

Dr. Spalding was aware of the difficulties spoken of, but did not regard them as insurmountable. Was never satisfied unless the cavity was kept dry during the operation of filling.

Dr. Morrison described a tongue holder which clamps under the chin. He had found it very useful in preventing the flow of saliva. Uses bibulous paper under the holder as a compress. When cavities extend below the margin of the gum, he would advise dissection of a portion of the gum sufficient to fully expose the cavity.

The use of vulcanized rubber as a base for artificial teeth was then introduced, Drs. McCoy, Spalding, Morrison, Peebles, Ellis and others, taking part in the discussion of this topic.

The general expression of sentiment showed that its use was regarded as disadvantageous both to the profession and the public, but as some cheap substance was demanded, its continued use was considered necessary.

The following resolutions were unanimously passed:

*Resolved*, That, in the opinion of this Society, the quality of the rubber now furnished by the manufacturers for dental purposes is greatly inferior to the early preparations of that material.

*Resolved*, That the President and Corresponding Secretary be requested to correspond with the India Rubber Company as to the cause of the deterioration of rubber prepared by them for dental use.

A short discussion was had on the importance of filling the deciduous teeth of children, for the purpose of preserving them till they were about to be succeeded by the permanent set.

Dr. Peebles wished to know the probable result in the following case, which occurred in his practice. In endeavoring to remove the pulp from a central incisor, preparatory to filling the root, the instrument used (a steel broach) was broken off in the canal of the root. Subsequently another and a larger instrument was broken off and left remaining in the root. The tooth was then filled with gold, leaving the instrument still in the root.

Dr. Spalding in reply, thought there was danger of galvanic action, which might cause pain by creating inflamation.

Dr. Forbes asked if the application of galvanism necessarily produces inflamation.

Dr. Abell had a similar case, and although he introduced a non-conducting substance between the two metals (steel and gold), the case became troublesome in two or three years. An examination showed the steel in a good state of preservation; refilled the crown without removing the steel from the root.

Dr. Rivers stated that a similar case had come under his observation. Patient complained of a frequent tingling sensation in the tooth. On extracting, he found the apex of root considerably absorbed and the steel point left projecting.

Dr. Spalding protests against filling over steel or other oxidisable metal. Has never failed to remove a broken instrument from the root canal.

During the evening session, the subject of sensitive dentine was introduced by Dr. McCoy, who wished to know the practice of members in treatment of such cases.

Dr. Forbes stated that he uses calcined alum; if that did not prove effective, he applied arsenious acid, provided the cavities were not deep.

Dr. Spalding had tried many things, among them Tinc. Benzoin and Tannic Acid, but he had found nothing that had produced satisfactory results. Thought that arsenious acid should be used with *great circumspection*, his observation convinced him that the death of the pulp was almost sure to follow its employment sooner or later. Had known it to occur two years or more after the arsenic had been employed.

Dr. Morrison recommended the Tinc. of Rad. Aconite.

Dr. Eames had used arsenic with bad results.

Dr. Ellis asked, in this connection, what caused the destruction of the floor of deep-seated cavities where the layer of dentine covering the pulp had become very thin.

It was thought to be caused by absorption of dentine resulting from inflammation of the pulp, induced by the introduction of the metalic filling in such close proximity to the pulp.

A vote of thanks was tendered to F. W. White, A. M., M. D. for his proposal, as editor and publisher of the St. Louis Medical and Surgical Journal, to publish the proceedings of this Society.

A vote of thanks was also given to the Board of Public Schools for the use of their very commodious room for the meetings of the Society.

